# Bioconversion of Glycosidic Precursors from Sour Guava (*Psidium friedrichsthalianum* Nied.) Fruit by the Oral Microbiota into Odor-Active Volatile Compounds

**DOI:** 10.3390/molecules27041269

**Published:** 2022-02-14

**Authors:** Carmen Tatiana Cuadrado-Silva, Carolina Muñoz-González, Ramón Giraldo, María Ángeles Del Pozo-Bayón, Coralia Osorio

**Affiliations:** 1 Departamento de Química, Universidad Nacional de Colombia, Bogotá DC AA 14490, Colombia; ctcuadrados@unal.edu.co; 2 Instituto de Investigación en Ciencias de la Alimentación (CIAL) (CSIC-UAM), C/Nicolás Cabrera, 9, Campus de Cantoblanco, 28049 Madrid, Spain; c.munoz@csic.es (C.M.-G.); m.delpozo@csic.es (M.Á.D.P.-B.); 3 Departamento de Estadística, Universidad Nacional de Colombia, Bogotá DC AA 14490, Colombia; rgiraldoh@unal.edu.co

**Keywords:** aroma precursors, glycosides, oral microbiota

## Abstract

The ability of the human oral microbiota to hydrolyze the glycosidic aroma precursor extract isolated from sour guava (*Psidium friedrichsthalianum* Nied.) fruits was studied herein. The glycosidic extract (GP) was incubated with a mixture of the oral microbiota isolated from three individuals’ saliva to evaluate the hydrolytic capacity of oral bacteria in the generation of odor-active compounds. The oral microbiota was able to release 1-hexanol from GP, under both aerobic and anaerobic conditions. Additionally, the aroma precursor extracts showed a decrease in the growth of harmful oral bacteria (*Streptococcus* and *Actinomyces*). This effect can be considered beneficial to human health because these bacteria have been related to different diseases of the bucco-respiratory tract.

## 1. Introduction

*Psidium friedrichsthalianum* Nied. is a tropical fruit that belongs to the *Myrtaceae* family. It is highly consumed in Central America and the Caribbean coast of South America. Its intense and pleasant aroma was characterized, identifying ethyl butanoate, (*Z*)-3-hexenal, ethyl hexanoate, and linalyl butyrate as key aroma compounds, as well as some sulfur compounds that were characterized as relevant odorants to its flavor [1]. This fruit has an intense aroma that somewhat resembles that of the common guava (*Psidium guava*), because ethyl butanoate, (*Z*)-3-hexenal, methional, 3-sulfanylhexyl acetate, and 3-sulfanyl-1-hexanol were also identified as *P. guava* odor-active volatiles. However, 4-methoxy-2,5-dimethyl-3(*2H*)-furanone, 4-hydroxy-2,5-dimethyl-3(*2H*)-furanone, 3-hydroxy-4,5-dimethyl-2(*5H*)-furanone, trans-4,5-epoxy-(*E*)-2-decenal, cinnamyl alcohol, ethyl butanoate, hexanal, methional, and cinnamyl acetate were identified as aroma-active compounds only in *Psidium guava* [2].

Aroma precursors have been widely studied in grapes and wine, mainly because of their role during the aging process in wine production [3]. During the 1980s and 1990s, several papers were published relating to the characterization of glycosidically bound volatiles and, hence, the influence of glycosides on the wine aroma by the effect of enzymatic or acid hydrolysis [4,5]. Since then, the related research has advanced, finding that flavors can also be released from odorless glycosides or amino acid conjugates by oral microbiota [6,7]. In this regard, it was first demonstrated, using one individual, that in-mouth hydrolysis of a simple glucoside can occur during tasting, together with the corresponding flavor perception [8]. Other sensory studies showed that phenol glycosides in smoke-affected wines can give a smoky retronasal aroma by in-mouth hydrolysis of the glycosides [9,10,11,12]. These studies indicated that whole fresh human saliva was able to release the smoky- and medicinal-smelling volatile phenols. More recently, Muñoz-González et al. [13] verified that the oral microbiota isolated from fresh saliva samples was able to hydrolyze odorless grape glycosides and release the corresponding volatile aglycones (terpenes, benzene derivatives, and C6-aliphatic compounds). This hydrolytic activity seems to be dependent on the bacteria present in the saliva, since it did not occur when the saliva was free of microorganisms (sterilized saliva). Later on, Parker et al. [14] showed the sensory significance of monoterpene glycosides during tasting by way of the retronasal perception of odorant aglycones released in the mouth and related to fruity flavors. Both works confirmed that the hydrolysis of glycosides and the production of odorant molecules is highly variable across individuals. Additionally, other oral processing parameters, such as the volume of the oral cavity, the dilution effect of saliva, and air flows, among others, can affect oral aroma release and, consequently, the in-mouth aroma perception [15].

There are no existing studies of the role of the oral microbiota on the hydrolysis of glycosidically bound volatiles in fruits during oral processing. Considering the relevance of precursors in the flavor of *P. friedrichsthalianum* Nied., the aim of this work was to determine the role of the oral microbiota in the generation of odor-active volatiles from glycosides. Therefore, the hydrolytic action of the oral microbiota over flavor precursors was evaluated in vitro, as well as the impact of these glycosides on the growth of oral microorganisms.

## 2. Results and Discussion

The *Psidium friedrichsthalianum* fruits were selected according to their ripeness, based on the values of the following parameters: pH (2.67 ± 0.06), soluble solid content (9.6 ± 0.3 °Bx), and titratable acidity expressed as citric acid percentage (2.75 ± 0.08%). Cuadrado-Silva et al. [1], by AEDA analysis of the SAFE extract of the whole fruits (without seeds), revealed the presence of 18 odor-active volatile compounds responsible for fruity, sweet, green, citrus, sulfury, and rancid-fermented odor notes. The volatile compounds with the highest FD values were ethyl butyrate, ethyl hexanoate, 3-sulfanylhexyl acetate, and *δ*-dodecalactone. Likewise, the role of glycosides as aroma precursors in *P. friedrichsthalianum* fruit was also studied by Cuadrado-Silva et al. [1], using a commercial glucosidase enzyme. The obtained extract was analyzed by GC–MS, finding that hexanol (562.4 mg/kg fruit) was the only odor-active volatile released from glycosidic precursors; this fact agrees with the presence of hexyl-*β*-D-glucopyranoside in this extract.

### 2.1. Impact of Oral Microbiota on Glycosidic Precursors (GP)

The oral microbiota is one the most complex bacterial communities associated with the human body; the SHI (bacteriological growth medium) culture medium was used because it is specific for this type of microorganism, very rich in nutrients, and it thus ensures the growth of a larger population of culturable oral bacteria [16]. The saliva samples of three volunteers were mixed because the variability of the oral microbiota of each individual greatly affects the hydrolysis of the aroma precursors [13].

In this experiment, only the microbiota isolated from raw saliva was used. To verify that bacteria from the oral cavity were able to hydrolyze the fruit glycosides under the established conditions, the standard octyl-*β*-D-glucopyranoside (20 ppm) was incubated as the positive control of the experiment. This glycoside was incubated separately under two conditions, aerobic (C1) and anaerobic (C2), and in both cases, the volatile compounds released were analyzed at four times, T1–T4 (0, 2, 24, and 72 h, respectively). The hydrolysis of this compound released 1-octanol under anaerobic conditions only at 24 h (2.09 ppm). This compound was identified by comparison of its MS and retention index (RI_DB-Wax_ = 1599) with those of the corresponding standard. Under aerobic conditions, 1-octanol was also detected, but at a lower intensity (not quantifiable). These results corroborate the hydrolytic capacity of oral cavity bacteria on glycosidic-type compounds, as already shown previously [13].

In the assays carried out with the GP extract from *P. friedrichsthalianum* fruit, 13 volatile compounds were detected after the incubation assays. These compounds were measured at four times, T1–T4, but in Table 1, only the data at initial time T1 are reported. Among the compounds, it seems that hexanol comes from the hydrolysis of glycosidic precursors because it was not detected in the control experiments without GP under anaerobic conditions (A3C2) and aerobic (A3C1) conditions. 

The volatile compounds found under anaerobic conditions in the A3 assay (only oral microbiota) were: 1-butanol, 3-methyl-1-butanol, acetic acid, 2-ethyl-1-hexanol, benzaldehyde, 4-butoxy-1-butanol, and 4-ethyl benzaldehyde; these may come from the decomposition of the SHI medium, which has a complex composition including peptone, trypticase peptone, yeast extract, sucrose, arginine, mucin, and sheep blood, among other constituents [16]. The mild conditions of incubation release a portion of these volatile compounds, but the amount was higher in the presence of oral microbiota. It was also determined that the highest production of volatile compounds was at 24 h when the oral microbiota was able to hydrolyze the glycosides; after that, the amount of volatile compounds started to decrease.

**Figure 1:**
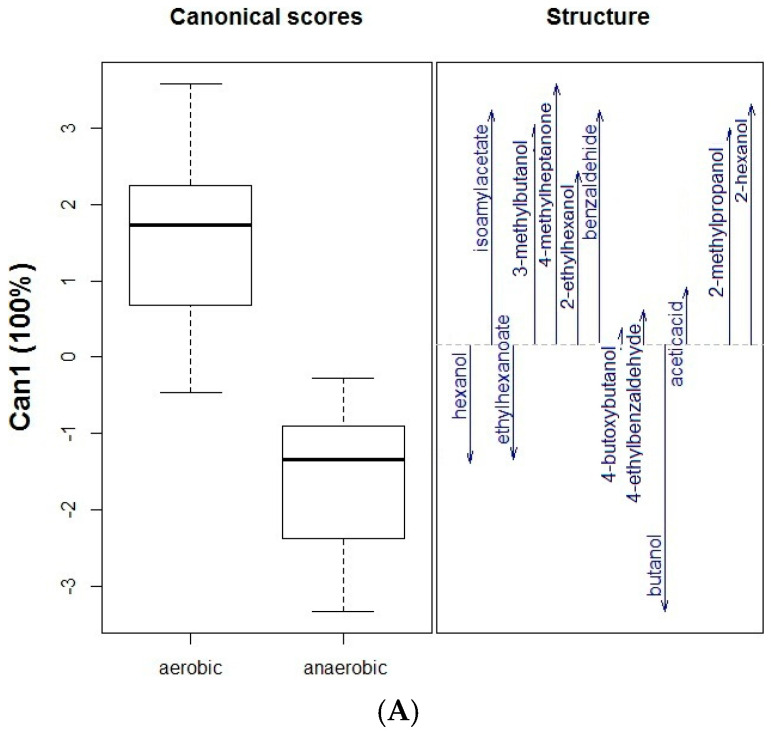

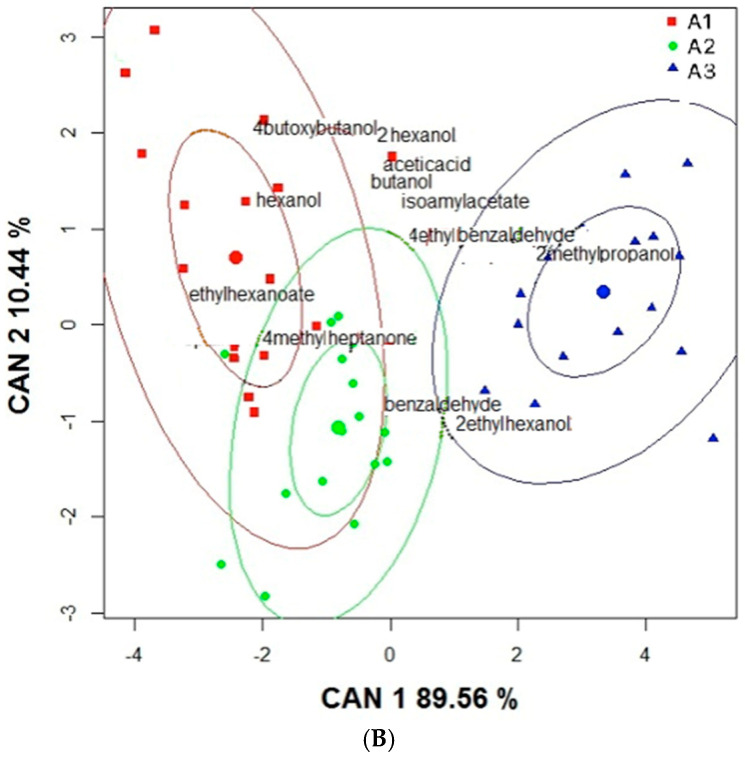
(**A**) Grouping of the volatile compounds obtained in the assays at different times (T1–T4) according to the incubation conditions (aerobic or anaerobic), by means of discriminant statistical analysis (DSA) by the square method. (**B**) Loading plot of linear discriminant analysis for volatile compounds found in different assays (A1, A2, and A3). A1 = GP incubation with oral microbiota; A2 = Only GP without oral microbiota; A3 = Only oral microbiota without GP.

Figure 1A,B show the results of discriminant analyses. Figure 1A used the conditions (aerobic or anaerobic) as a qualitative variable and shows that high amounts of hexane, ethyl hexanoate, and butanol were produced under anaerobic conditions. In contrast, higher amounts of the other compounds were obtained under aerobic conditions. This finding suggests that hydrolysis under anaerobic conditions is more effective in producing odorant compounds. On the other hand, Figure 1B used the assay (A1, A2, or A3, the meaning of which is explained in the footnote of Table 1) as a qualitative variable. According to Figure 1B, there is an overlap between the A1 and A2 assays; namely, there are similar amounts of some of the compounds in both assays (i.e., 4-methyl-2-heptanone). In the assay A1 (GP + OM), high values (above the mean) of hexanol and 4-butoxy-butanol were obtained. Additionally, above-average amounts of benzaldehyde, 2-ethyl-hexanol, and 2-methyl-propanol were obtained in the A3 assay (only oral microbiota). The volatiles released during different hydrolysis assays could be grouped into three clusters, showing that hexanol and ethyl hexanoate were only present in the A1 experiment. The volatile compounds with hydroxyl groups are usually linked to sugars, but carboxylic acids can also be linked as glycosides. This could be an explanation for the presence of an ester in this experiment.

**Figure 2:**
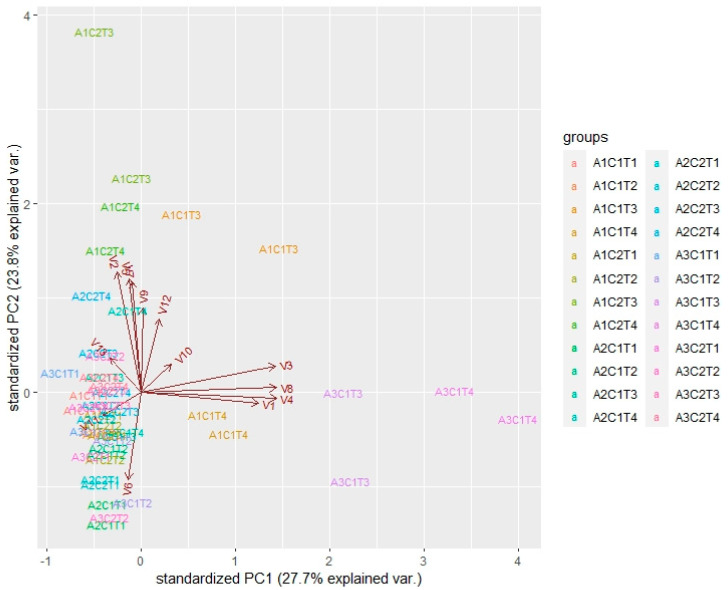
Principal component analysis (PCA) results based on the data of 13 volatile compounds released, using as supplementary variables the factors of assay (A1, A2, A3), experimental conditions (C1, C2), and time (T1, T2, T3, T4). V1 = 2-Methyl-propanol; V2 = butanol; V3 = 2-hexanol; V4 = 3-methyl-butanol; V5 = hexanol; V6 = 2-ethyl-hexanol; V7 = 4-butoxy-butanol; V8 = isoamyl acetate; V9 = ethyl hexanoate; V10 = 4-methyl-2-heptanone; V11 = benzaldehyde; V12 = 4-ethyl benzaldehyde; V13 = acetic acid. The labels used are the same as those in Table 1.

Table S1 in the Supplementary Material shows (as an example of what happens with the 13 volatiles) the results of a three-way analysis of variance carried out with the values of 2-methyl-propanol and taking as factors the assay (three levels), experimental conditions (two levels), and time (four levels). A remarkable aspect is an interaction between the factors (assay, condition, and time). Similar results (not shown to save space) were obtained with the other 12 volatile compounds. This situation indicates that it is convenient to complement discriminant analyses with others that help to visualize in an integrated way the variability of the compounds concerning the combination of the levels of the three factors (assay, condition, and time of hydrolysis). To this end, a principal component analysis (PCA, Figure 2) was carried out with all available information, to analyze the relationship between the compounds and factors simultaneously. 

Figure 2 shows the results regarding the two first components, which explain 51.5% of the variability of the analyzed data. The most notable results are interpreted as follows. The records of assay A1 (GP+OM), under anaerobic (C2) conditions, at times T3 and T4 (24 and 72 h), are associated with values above the average in butanol (V2) and hexanol (V5), indicating that these alcohols are produced by hydrolysis of glycosidic precursors by the oral microbiota. 4-Butoxy butanol (V7), ethyl hexanoate (V9), and 4-ethyl benzaldehyde (V12) also showed high values under both conditions (C1 and C2) but they showed interaction in the three-way analysis of variance (data not shown) that did not allow us to draw any conclusion. Ethyl hexanoate (V9) was present in significant amounts in the A1C1T3 and A2C2T3 assays, which means that it was present in GP extract without hydrolysis by oral microbiota. The PCA clearly shows that the compounds with values above the average in assay A3—2-hexanol (V3), isoamyl acetate (V8), 3-methyl-butanol (V4), and 2-methyl-propanol (V1)—were produced by the oral microbiota under aerobic (C1) conditions at times T3 and T4 (24 and 72 h). Finally, 2-ethyl-hexanol (V6) and benzaldehyde (V11) did not show preferable conditions for production. 

### 2.2. Effect of P. friedrichsthalianum Glycosidic Precursors on the Oral Microbiota

**Figure 3:**
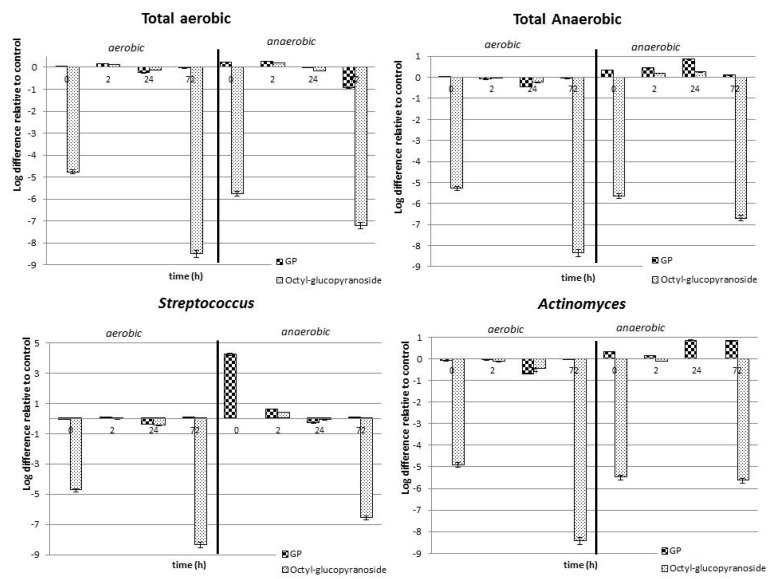
Changes in bacterial group populations during incubation of GP extract and octyl-*β*-D-glucopyranoside with the human oral microbiota of saliva from three individuals. The control was a bacterial population without GP. Total aerobic and anaerobic bacteria counts were 62,000 and 240,000 CFUs/mL, respectively.

The interaction between *P. friedrichsthalianum* glycosidic precursors and the oral microbiota is a two-way interaction because the microbiota is important for aroma release, while glycosides enhance the beneficial bacteria in the mouth. Thus, the GP extract did not cause any increase in the bacterium population during the total incubation time (72 h) under aerobic conditions; in contrast, the GP extract produced an increase in the counts of total anaerobic bacteria and *Actinomyces* with respect to the growth control. This behavior is opposite to that observed for *Streptococcus*: their population decreased during the incubation time (Figure 3). The octyl-*β*-D-glucopyranoside produced a significant decrease in the bacterium population, under both aerobic and anaerobic conditions. This is evident in the bars, since there are large and negative values, indicating a lower population for the bacteria incubated with octyl-*β*-D-glucopyranoside in comparison with when the bacteria were alone.

These results indicate a decrease in the growth of human oral cavity bacteria after in vitro hydrolysis of glycosidic precursors, suggesting a role of the released volatile compounds. In this study, the microbiota composition was not characterized by molecular techniques; however, the growth conditions employed and the use of a culture medium specific for representative bacteria of the oral cavity allowed us to characterize the main groups as follows. *Staphylococcus aureus* and *Enterococcus faecalis* are representative of the total aerobic bacterial species, while *Granulicatella adiascens*, *Veillonella dispar*, and *Fusobacterium nucleatum* are the corresponding for total anaerobes; in addition, *Streptococcus sanguinis* is the most representative species within *Streptococcus* of the oral cavity, whereas *Actinomyces naeslundii* is one of the most representative among *Actinomycetes* [13]. It is known that the genus *Streptococcus* is a group of Gram-positive coccus bacteria belonging to the Firmicutes family and to the group of lactic acid bacteria. Depending on the species, some of them have been linked to oral cavity and respiratory tract diseases, such as tonsillitis, pneumonia, and dental caries. The genus *Actinomyces* corresponds to Gram-positive bacteria. Some species are anaerobic, while others are facultative anaerobes. Many *Actinomyces* are opportunistic pathogens of humans and other mammals, particularly in the oral cavity; in some cases, these bacteria can cause actinomycosis, a disease characterized by the formation of abscesses in the mouth, lungs, or gastrointestinal tract [17].

Both the GP of the fruits of *P. friedrichsthalianum* and the released aglycones can reduce the growth of some of the bacteria in question under in vitro conditions. It has been reported that this type of bacteria easily adapts to in vivo conditions and can recolonize clean surfaces in the mouth in a few minutes, due to the formation of biofilms on the teeth. *Streptococcus* and *Actinomyces* are the first settlers of the dental biofilm [18]. Thus, these results open the possibility of new studies related to the possible prevention of some bucco-respiratory tract diseases related to the oral microbiota by the consumption of *P. friedrichsthalianum*.

## 3. Materials and Methods

### 3.1. Materials

Dichloromethane, pentane, methanol, sodium sulfate (anhydrous), sodium chloride, ammonia (solution 25% *w*/*w*), sodium hydroxide, 3-methyl-1-butanol, acetic acid, 1-octanol, and n-alkane mix (C_8_–C_26_) were acquired from Merck (Darmstadt, Germany). Peptide protease, urea, mucin (Type III), and NAM (N-acetylmuramic acid) were acquired from Sigma-Aldrich (Steinheim, Germany). Defibrillated lamb blood was supplied by Dismolab (Madrid, Spain). Pure water was obtained from a Milli-Q purification system (Millipore, Bedford, MA, USA). The following compounds were commercially acquired and used as references: octyl-*β*-D-glucopyranoside, ethyl hexanoate, 3-octanol, and butanoic acid from Sigma-Aldrich (Steinheim, Germany); hexanol and 3-sulfanyl-1-hexanol from Alfa Aesar (Heysham, UK); and benzaldehyde and 2-methyl propanoic acid, generously supplied by DISAROMAS S.A (Bogotá, Colombia). Bacteriological growth medium (SHI medium) was purchased from Oxoid Ltd. (Basingstoke, UK); it contained peptone protease (10 g/L) (Difco), peptone trypticase (5 g/L) (Difco), yeast extract (5 g/L) (Difco), KCl (2.5 g/L), sucrose (5 g/L), hemin (5 mg/L), vitamin K (1 mg/1μL), urea (60 mg/L), arginine (0.174 g/L), mucin (type III) (2.5 g/L), lamb’s blood defibrillated at 5% (50 mL/L), NAM (N-acetylmuramic acid) (10 mg/L), and distilled water [16]. Sterilization of culture media and microbiology materials and instruments was carried out in an autoclave (brand: BMT, model: Sterivap hp il, Brno, Czech Republic) at 121 °C for 15 min.

### 3.2. Plant Material

Ripe fruits were acquired from local markets of Montería (Córdoba, Colombia), and they were selected according to their ripeness qualities. Titratable acidity was determined in triplicate by using 10 g of fruit pulp, following the procedure published by AOAC [19], and the results are expressed as a percentage of citric acid. The pH of the pulp was determined by using a Jenway pHmeter (model 370, Essex, UK). Total soluble solids were determined using an Atago refractometer (HRS-500, Tokyo, Japan), and the results are expressed in degrees Brix (°Bx).

### 3.3. Preparation of Human Saliva Samples

Samples of stimulated saliva were collected from three different individuals (all female), aged 27–42 years. All volunteers were in good health, without any particular dietary condition, and non-smokers, and they had not used antibiotics in the six months prior to the study. The samples were collected at the site on the day of the experiment and used immediately. Before collecting samples, the volunteers brushed their teeth without toothpaste or mentholated mouthwash and did not eat or drink anything one hour before collecting saliva.

Saliva samples were collected per individual (15 mL); then, the three individual saliva samples were homogenized, mixed, and finally centrifuged at 4500 rpm for 10 min at 4 °C. The supernatant was diluted 1:5 (*v*/*v*) with the SHI medium and homogenized, and the homogenate was divided into two parts to be incubated at 37 °C overnight: one under aerobic and the other under anaerobic conditions.

### 3.4. In Vitro Experiments with the Glycosidic Extract (GP) and Human Saliva

The glycosidic precursor extract (GP, 4.1 g/Kg fruit) from *P. friedrichsthalianum* was obtained following the procedure reported by Cuadrado-Silva et al. [1] by selective adsorption of fruit polar extract on Amberlite XAD-4. The experiments were set up by placing 24 mg of GP with 12 mL of saliva inoculum (2 mL of saliva mixture/10 mL of SHI medium) in 50 mL sterile falcon tubes [16]. Additionally, three different control experiments were performed: (A) incubation of the oral microbiota in the medium, but without the GP, to evaluate the volatile compounds derived from basal metabolism (A3); (B) incubation of the GP in the medium but without the oral microbiota, to determine the changes due to chemical transformation under the conditions of the incubation medium (A2); and (C) incubation of the positive control, using octyl-*β*-D-glucopyranoside (20 ppm), the SHI medium, and oral bacteria, to verify the hydrolytic activity of bacteria by releasing 1-octanol. All the cultures were made at 37 °C for a 72 h period, with constant agitation at 140 rpm, in duplicate. A 4 × 2 random experimental design was performed, with four sample collection times that were representative of the original sample (0 h, T1), the initial stage of hydrolysis (2 h, T2), the hydrolysis time (24 h, T3), and the post hydrolysis time (72 h, T4), and two microbial growth conditions: aerobic (C1) and anaerobic (C2). At each time, 2 mL samples were collected in glass vials (for HS-SPME, 20 mL volume); these were used for the analysis of volatile compounds by GC–MS. Likewise, samples were taken in sterile Eppendorf tubes (1.5 mL) at the indicated times and stored with aqueous 40% glycerol (1:1) at −70 °C.

### 3.5. HS-SPME (Headspace Solid-Phase Microextraction) of Volatile Compounds after Incubation of the P. friedrichsthalianum Aroma Precursors with Human Saliva

The procedure was adapted from that described by Muñoz-González et al. [13]. In vials, 40 μL of the internal standard for quantification solution (3-octanol 10 ppm) was added to the sample (2 mL), as well as 0.5 g of NaCl. The extraction procedure was automatically performed using a CombiPal system (CTC Analytics AG, Zwingen, Switzerland) with a 2 cm long DVB/CAR/PDMS fiber (Supelco, Bellefonte, PA, USA). Samples were preincubated for 10 min at 35 °C, and the extraction was done in the headspace of the vial for 5 min at 35 °C. The desorption was performed in a gas chromatograph injector (Agilent 6890N, Agilent, Palo Alto, CA, USA) in splitless mode for 90 s at 270 °C. After each injection, the fiber was cleaned for 10 min to remove any residual volatile compounds. To quantify the compounds, the internal standard method was used.

### 3.6. GC–MS (Gas Chromatography–Mass Spectrometry) Analysis of Volatile Compounds

The GC–MS analyses were performed on an Agilent 6890N gas chromatograph coupled to an Agilent 5973N mass spectrometer (Agilent, Palo Alto, CA, USA), operated in electronic impact ionization mode (EI) 70 eV; the positive ions with masses between 40 and 350 u were detected. A DB-WAX capillary column (60 m, 0.250 mm, 0.50 μm, Agilent, Palo Alto, CA, USA) was used, with the following temperature program: the oven was maintained for 5 min at 40 °C, and then the temperature was increased at 4 °C/min until 240 °C, where it was finally maintained for 20 min. The temperature was 270 °C for the injector and 230 °C for the ion source. In all of the cases, the injections were made in splitless mode, using helium (UAP grade) as a carrier gas at a rate of 1 mL/min. The data were processed in the Agilent ChemStation software. The identification of the compounds was carried out by comparison of their retention index and mass spectra with those of the corresponding reference substances; if they were not available, the identification was done by comparison of their retention indices and the mass spectra with those reported in the NIST 2.0 mass database. Quantitation of volatile compounds was performed by comparison of the areas with that of the standard (3-octanol, 10 ppm, RI_DB-Wax_ = 1429).

### 3.7. Counting of Microorganisms in Human Saliva

The samples stored in glycerol were serially diluted by using saline normal solution (NaCl 0.9% in water). The number of total colony-forming units (CFU/mL) of each type of bacteria was quantified by counting colonies after direct spot-seeding of plate dilutions of four medium types: TSB-Agar (BD, Franklin Lakes, NJ, USA) for total aerobes; Wilkins-Chalgren agar (BD, Franklin Lakes, NJ, USA) for total anaerobes; TBS-Agar modified with 0.3% yeast extract (BD, Franklin Lakes, NJ, USA) for Streptococcus; and BHI-Agar modified with 1% casein, 0.5% glucose, and 0.5% yeast extract (BD, Franklin Lakes, NJ, USA) for Actinomyces. The plates were incubated at 37 °C for 48 h under anaerobic conditions (Bactron Anaerobic/Environmental Chamber, SHELLAB, Cornelius, OR, USA), except for TSB-Agar plates, which were incubated under aerobic conditions.

### 3.8. Statistical Analysis

Initially, discriminant analysis was carried out to establish the relationship of the released compounds with the three assays (A1, A2, and A3) or the presence of oxygen (C1 and C2). A three-way ANOVA was performed for each compound in the second instance, taking assay, time, and conditions as factors. These analyses allowed us to determine whether there was an interaction between the factors, i.e., if the differences between the levels of the factors can be identified separately or if they need to be taken into account together. Subsequently, a principal component analysis (PCA) was used to jointly establish the interrelationship of the compounds with the three factors of interest from a descriptive perspective. The following programs were used for statistical data processing and graphics: RWIZARD and RStudio for Windows (version 1.1), and Microsoft Office EXCEL for Windows (version 2010). Differences at probability level *p* ≤ 0.05 were considered significant. The levels were: 3 assays (A1, A2, and A3); 2 conditions related to the presence of oxygen (C1 and C2: aerobic and anaerobic conditions, respectively); 4 incubation times (T1, T2, T3, and T4: 0, 2, 24, and 72 h, respectively); and the 13 volatile compounds (V1–V13, see Table 1). All analyses were performed in duplicate. 

## 4. Conclusions

The reported results show that the oral cavity microbiota from human saliva has hydrolytic activity on the glycosides present in *Psidium friedrichsthalianum* Nied., which was evidenced by the detection of 1-hexanol and butanol after incubation of the glycosidic precursor extract (GP) with the cultivable bacteria from several healthy individuals. Although this study is far from consumption conditions, the existence of this hydrolytic activity could be a relevant mechanism in the generation of new odorant molecules when sour guava (*P. friedrichsthalianum*) is consumed, and this could contribute to the perception of its aroma and acceptability. Future research should aim to evaluate the effect of the oral microbiota on aroma precursors and off-taste notes in *P. friedrichsthalianum* fruit using in vivo conditions.

## Figures and Tables

**Table 1:** Volatile compounds detected at the initial time (t = 0 h, T1) of the incubation of *P. friedrichsthalianum* glycosidic extract with oral microbiota isolated from saliva under different conditions.

No.	Compound ^a^	RI_DB-wax_	Aerobic Conditions (C1, Amount in ppm) ^e^			Anaerobic Conditions (C2, Amount in ppm) ^e^		
			A1	A2	A3	A1	A2	A3
V1	2-Methyl-1-propanol ^b^	1085	0.00315	0.00168	0.00297	0.00178	0.00064	-
V2	1-Butanol ^b^	1176	0.02510	0.01480	0.03870	0.02450	0.01400	0.03430
V3	2-Hexanol ^b,d^	1197	0.05350	-	0.05800	0.01510	-	-
V4	3-Methyl-1-butanol	1213	0.03060	0.00476	0.05430	0.01620	0.00406	0.02960
V5	Hexanol ^c,d^	1368	0.02200	0.03800	-	0.19100	-	-
V6	2-Ethyl-1-hexanol ^b^	1494	0.14400	0.20400	0.15800	0.13100	0.14500	0.13700
V7	4-Butoxy-1-butanol ^b^	1691	0.01680	0.00707	0.01270	0.01360	0.00874	0.00944
V8	Isoamyl acetate ^b,d^	1159	0.02500	-	0.03220	-	-	-
V9	Ethyl hexanoate ^c^	1238	0.00592	0.00304	-	0.00877	0.00207	-
V10	4-Methyl-2-heptanone ^b^	1250	0.00898	0.00805	-	0.00550	0.00269	-
V11	Benzaldehyde	1528	0.42000	0.14800	0.54000	0.23900	0.16500	0.25000
V12	4-Ethyl benzaldehyde ^b^	1786	0.02020	0.01430	0.02860	0.01960	0.01450	0.02200
V13	Acetic acid	1452	0.08790	0.01740	0.10900	0.01150	0.01920	0.05060

A1 = Incubation of the GP in the medium with oral microbiota (GP + OM); A2 = Incubation of the GP in the medium without oral microbiota (only GP); A3 = incubation of the medium with saliva (only OM). ^a^ Volatile compounds released were identified by comparing their retention indices (RI) on the DB-Wax column and their mass spectra with respective data of reference compounds. ^b^ Identified only by comparison with the NIST 2.0 mass database and PubChem database. ^c^ Odor-active volatiles in *P*. *friedrichsthalianum* fruit [1]. ^d^ Compounds detected after 24 h of incubation (T3), not detected at the initial time (T1). ^e^ All the analyses were performed in duplicate, and 3-octanol was used as an internal standard. - = not detected.

## Data Availability

The data presented in this study are available on request from the corresponding author.

## Supplementary Materials

The following supporting information can be downloaded online, Table S1: Analysis of variance from data of 2-methyl-propanol obtained under the levels of three factors (assay, condition, and time).

## Sample Availability

Samples of the volatile compounds and microbiota of saliva are no longer available.
