# Supplementary material for: Bioconversion of Glycosidic Precursors from Sour Guava (Psidium friedrichsthalianum Nied.) Fruit by the Oral Microbiota into Odor-Active Volatile Compounds
Source: Molecules. 2022 Feb 14;27(4):1269. doi: 10.3390/molecules27041269 (PMC8875828; doi:10.3390/molecules27041269)
Supplement: Supplementary file 1 [file molecules-27-01269-s001.zip › molecules-1558752-supplementary.pdf]

Table S1. Analysis of variance from data of 2-methyl-propanol obtained under the levels of three factors (assay, condition, and time)

Factor	*DF*	*Sums of squares*	*Mean Squares*	*F value*	*Pr(>F)*
Assay	*2*	0.0004436	2.218 e-04	21.726	4.12e-06
Condition	*1*	0.0002446	2.446 e-04	23.958	5.43e-05
Time	*3*	0.0001793	3 5.976 e-05	5.854	0.00378
Assay-Condition	*2*	0.0002904	1.452 e-04	14.226	8.42e-05
Assay-Time	*6*	0.0003221	5.369 e-05	5.259	0.00139
Condition-Time	*3*	0.0000834	2.779 e-05	2.722	0.06670
Assay-Condition-Time	*6*	0.0002078	3.463 e-05	3.392	0.01445
Residuals	*24*	0.0002450	1.021 e-05		
